# Evaluation of COPAN FecalSwab and eSwab collection systems for the detection of C*lostridioides difficile* using the BD MAX Cdiff assay

**DOI:** 10.1128/spectrum.00767-26

**Published:** 2026-05-01

**Authors:** Kelly Waters, Keltie Baldwin, Sarah Marttala, Doris Williams, David Bulir, Mohammad Rubayet Hasan, Marek Smieja, Jodi Gilchrist

**Affiliations:** 1 Research St. Joseph’s – Hamilton 539740 , Hamilton, Ontario, Canada; 2 Department of Chemical Engineering, McMaster University 3710, Hamilton, Ontario, Canada; 3 Department of Pathology and Molecular Medicine, McMaster University 3710, Hamilton, Ontario, Canada; 4 Hamilton Regional Laboratory Medicine Program, St Joseph's Healthcare Hamilton 25479, Hamilton, Ontario, Canada; 5 Department of Health Research Methods, Evidence, and Impact, McMaster University 3710, Hamilton, Ontario, Canada; Johns Hopkins University, Baltimore, Maryland, USA

**Keywords:** polymerase chain reaction, *Clostridioides difficile*, specimen collection

## Abstract

*Clostridioides difficile* infection (CDI) requires accurate and timely diagnostic testing to guide treatment. Nucleic acid amplification tests (NAATs) offer high sensitivity and specificity, but most assays, including the Health Canada-approved BD MAX Cdiff assay, are validated only for unpreserved stool. This presents challenges for transport, stability, and specimen sharing between tests. Swab-based collection systems such as COPAN FecalSwab and eSwab may alleviate these limitations but have not been fully evaluated on the BD MAX Cdiff assay. Residual stool specimens submitted for CDI testing were used to prepare FecalSwab and eSwab aliquots and evaluated to determine the optimal volume for BD MAX Cdiff testing. Sensitivity, specificity, and agreement were calculated using unpreserved stool as the reference method. Stability of *C. difficile* DNA was assessed at 4°C, room temperature, and 37°C over 14 days. Minimal variation in *C. difficile* PCR cycle threshold (Ct) values was observed across varying volumes of FecalSwab and eSwab media, and 100 µL was selected. *C. difficile* DNA was detected in all specimen types up to 14 days at different temperatures. However, a gradual increase in Ct values was noted over time for all specimens and storage temperatures, except for FecalSwab specimens stored at 4°C. FecalSwab specimens demonstrated 98.5% sensitivity and 98.4% specificity, while eSwab specimens demonstrated 97.6% sensitivity and 100% specificity against unpreserved stool. Agreement between the two swab types was 98.4%. FecalSwab and eSwab specimens prepared from unpreserved stool demonstrate excellent agreement with unpreserved stool, and provide improved options for specimen transport and stability.

**IMPORTANCE:**

This study demonstrates the utility of FecalSwab and eSwab collection media on the BD Max Cdiff assay. This addresses an important gap in the diagnosis of *Clostridioides difficile* infection by evaluating other specimen types beyond neat stool specimens. We demonstrate that swabs collected from stool into FecalSwab and eSwab media have excellent sensitivity and specificity compared to neat stool. Further, FecalSwab and eSwab media allow for preservation of *C. difficile* DNA at varying storage conditions. This could alleviate specimen transport challenges in remote or resource-limited settings.

## INTRODUCTION

*Clostridioides difficile* infection (CDI) is a global public health concern that commonly causes fever, abdominal pain, cramping, and frequent watery diarrhea. Severe infection can lead to serious dehydration, toxic megacolon, and even death. The pathogenesis of *C. difficile* disease is mediated by the production of toxins A and B, which disrupt the intestinal lining and initiate an inflammatory process in the bowels (1). Effective management of *C. difficile* infection relies on accurate, timely diagnosis to facilitate appropriate and rapid treatment. Diagnosis is made by the detection of *C. difficile* toxin or DNA in stool specimens from symptomatic patients. Most current diagnostic tests require a liquid stool sample (1, 2), but unpreserved stool can present challenges for sample transport and stability.

Stool culture for detection of toxigenic *C. difficile* is highly sensitive but labor-intensive, not widely available, and may require up to 96 h to provide results (3). Antigen/enzyme immunoassay methods targeting *C. difficile* glutamate dehydrogenase (GDH) and toxins can produce rapid results with limited training but have variable sensitivity and specificity (4, 5). Nucleic acid amplification tests (NAATs), such as polymerase chain reaction (PCR) and loop-mediated amplification (LAMP), have high sensitivity and specificity but often require specialized equipment and technical training. Current Infectious Disease Society of America (IDSA) guidelines for the diagnosis of symptomatic *C. difficile* recommend the use of a NAAT alone or in combination with a GDH plus toxin test on unpreserved stool (2). The BD MAX Cdiff PCR assay has been shown to have a sensitivity of 88–95% for detecting *C. difficile* from unpreserved stool in clinical practice (6, 7).

Swabs collected from neat stool could be a valuable alternative to conventional stool collection and transport, as swab collection tubes and preservation media could alleviate sample transport and stability challenges. Additionally, *C. difficile* testing of swabs collected from unpreserved stool provides an option for specimen sharing with other PCR assays for gastrointestinal pathogens. It has previously been demonstrated that COPAN FecalSwab specimens collected from neat stool in Cepheid’s Xpert *C. difficile* assay yielded equivalent results and high positive and negative percent agreements with standard methods (8). Additionally, Hirvonen et al. demonstrated lower rates of PCR inhibition in FecalSwab specimens when compared with unpreserved stool specimens when using both the GenomEra *C. difficile* and BD MAX Cdiff assays (9). An additional study showed good agreement with a reference laboratory’s GDH and toxin algorithm when using FecalSwabs in the BD MAX Cdiff assay (10). Overall, FecalSwab specimens have been shown to perform well on multiple *C. difficile* assay platforms.

An additional commonly available specimen transport medium is the COPAN eSwab medium. While eSwab collection devices are well-utilized as rectal swabs for methicillin-resistant *Staphylococcus aureus* (MRSA) and vancomycin-resistant enterococci (VRE) screening, little work has been done on their utility for *C. difficile* testing from a stool sample. However, FecalSwab and eSwab collection kits were compared for *C. difficile* viability and survival at a variety of storage conditions, with FecalSwab performing better than eSwab at lower temperatures while both showed comparable recoveries at room temperature (11).

The BD MAX platform is a fully automated, random access instrument that offers rapid detection of *C. difficile* with limited hands-on time and high sensitivity and specificity. BD MAX Cdiff assay is based on the detection of *C. difficile* toxin B gene (tcdB), and the assay is approved by Health Canada and the United States Food and Drug Administration (FDA) for use with unpreserved, liquid stool. We hypothesized that the use of FecalSwab or eSwab specimens prepared from unpreserved stool samples for BD MAX Cdiff testing would result in equivalent test accuracy and comparable *C. difficile* DNA stability. FecalSwab or eSwab collection kits paired with an assay that is faster and easier to perform could reduce the time to result and time to treatment. Therefore, in this study, we evaluated the performance of FecalSwab and eSwab specimens collected from unpreserved stool samples for use with the BD MAX Cdiff assay and assessed the stability of *C. difficile* DNA in the collection media under various storage conditions.

## MATERIALS AND METHODS

**Fig 1:**
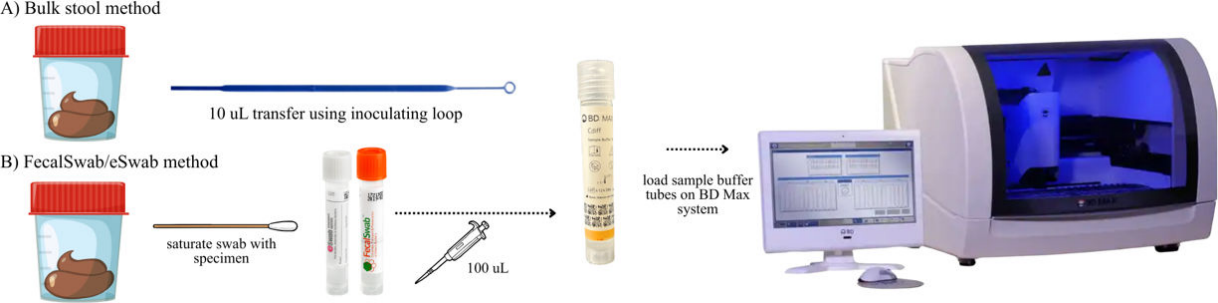
Specimen preparation workflow for neat stool (**A**), and FecalSwab and eSwab (**B**) specimens.

Residual bulk, unpreserved stool specimens (*n* = 127) were retrospectively collected in dry stool containers and submitted to the Hamilton Regional Laboratory Medicine Program (HRLMP) for standard of care *C. difficile* testing. These include specimens that tested positive or negative for *C. difficile* by an in-house developed and validated LAMP assay (data not shown). All specimens met HRLMP’s criteria for *C. difficile* testing, which included both liquid and solid stools. All specimens were accessed and tested in the BD MAX within 24 h of collection. FecalSwabs and eSwabs of unpreserved stool were prepared by inserting the swab into the stool specimen, saturating the swab, returning to the screwcap tube, and vortexing for 10 s (Fig. 1). FecalSwabs and eSwabs of stool were also prepared within 24 h of collection.

Initially, to determine the optimal volume of each medium, varying volumes of FecalSwab and eSwab medium (10, 50, 100, 200, and 400 uL) were transferred to the BD sample buffer tube and tested on the BD MAX Cdiff assay in triplicate against the manufacturer-recommended volume of 10 uL of unpreserved stool. After selecting the optimal volume, the sensitivity and specificity of FecalSwab and eSwab were calculated using unpreserved stool as the gold standard, and statistical agreement was assessed using Cohen’s kappa coefficient. Finally, temperature and time stability of specimens collected in FecalSwab and eSwab media were assessed at 4°C, room temperature, and 37°C for 48 h, 7 days, and 14 days. For each specimen type, change in Ct values (ΔCt) was measured from day 0 Ct value obtained for the respective specimen and plotted against time. To evaluate ΔCt trends over time and compare swab types, we fitted linear models using R (version 2024.12.1+563), including interaction terms between day and swab_type: "ΔCt"∼"day"*"swab_type". The main effect of day representsthe slope (change in ΔCt per day) for the reference swab type (unpreserved stool). Interaction terms (day:swab_type) test whether the rate of change over time differs between FecalSwab or eSwab and unpreserved stool. Significance of model terms was assessed using standard *t*-tests, with *P* values < 0.05 considered statistically significant. Model fit was evaluated using *R*^2^ and residual diagnostics.

## RESULTS

### Determination of optimal volume of FecalSwab and eSwab specimens for *C. difficile* testing

Different volumes of FecalSwab and eSwab specimens were prepared in triplicate from a *C. difficile*-positive stool sample and tested using the BD MAX Cdiff assay. The Ct values obtained for the *tcd*B gene were compared with those obtained from the manufacturer-recommended unpreserved stool testing (Table 1). While all volumes showed minimal change in Ct value compared to that of the manufacturer-recommended unpreserved stool volume, overall, 100 µL was selected as the optimal volume of swab specimens to proceed with assessing sensitivity and specificity based on limited variability in replicate testing, minimal change in Ct values, and lab workflows.

**TABLE 1:** Comparison of *tcd*B Ct values from different volumes of FecalSwab and eSwab specimens with those from 10 µL of neat stool in the BD MAX Cdiff assay

Specimen volume	Mean neat stool Ct (±SD)	Mean FecalSwab (Ct) (±SD)	FecalSwab (ΔCt)	Mean neat stool (Ct) (±SD)	Mean eSwab (Ct) (±SD)	eSwab (ΔCt)
10 µL	26.47 (1.19)	27.43 (0.75)	0.96	27.87 (0.46)	28.77 (0.32)	0.90
50 µL	N/A^*a*^	26.7 (0.46)	0.23	N/A	28.1 (0.35)	0.24
100 µL	N/A	26.2 (0.75)	−0.27	N/A	27.47 (0.25)	−0.40
200 µL	N/A	25.93 (0.90)	−0.54	N/A	28.47 (0.40)	0.6
400 µL	N/A	26.67 (0.75)	0.20	N/A	27.73 (0.45)	−0.13

^*a*^ N/A, not applicable.

### Determination of optimal storage and transport conditions for FecalSwab and eSwab specimens for *C. difficile* testing

Three distinct *C. difficile*-positive specimens were used to prepare FecalSwab and eSwab specimens, which were aliquoted and stored at 4°C, room temperature, and 37°C for 14 days. Aliquoted unpreserved stool was stored alongside the swabs as a control. BD MAX C. diff testing was performed on days 0, 2, 7, and 14.

**Fig 2:**
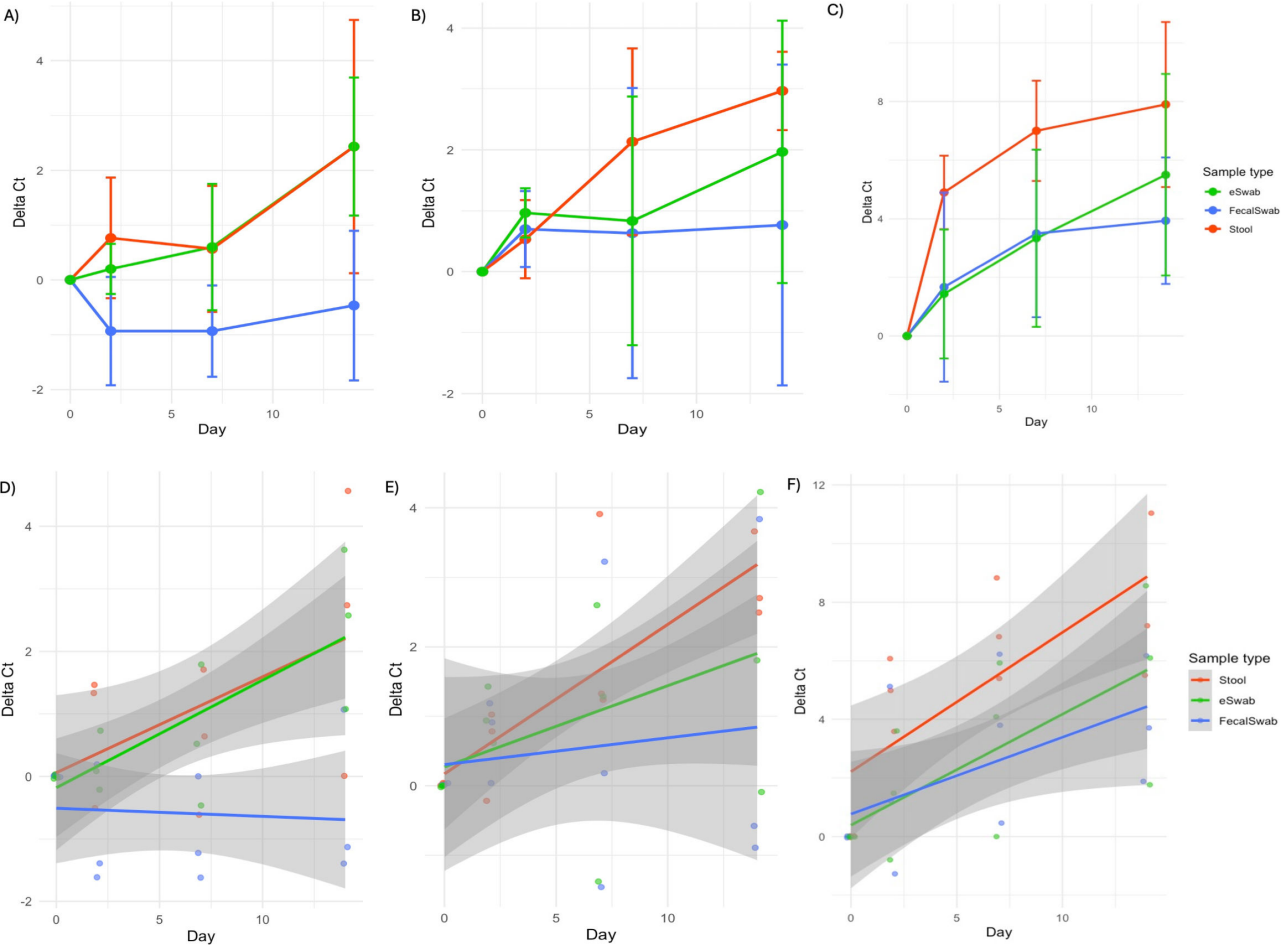
Stability of *C. difficile* DNA in unpreserved stool, FecalSwab, and eSwab specimens over 14 days at 4°C (**A and D**), room temperature (**B and E**), and 37°C (**C and F**). Panels **A** through **C** show the mean changes in *C. difficile* Ct values (ΔCt) over time. Error bars show standard deviation. Panels **D** through **F** present scatterplots of ΔCt versus day, generated with ggplot2, with linear regression lines and 95% confidence intervals overlaid to illustrate trends for each swab type.

Figure 2A through C shows the mean change in Ct over time for each storage condition and specimen type. Under all three conditions, the smallest change in Ct value was observed in FecalSwab specimens stored at 4°C. Separate one-way ANOVAs were conducted at each temperature and timepoint to compare ΔCt values between sample types. No statistically significant differences were observed for any of the conditions tested (all *P* > 0.05). Tukey’s HSD post hoc analyses confirmed the absence of significant pairwise differences. Levene’s test was non-significant in all cases (*P* > 0.05), indicating that the assumption of equal variances was met (Table S1).

We next compared the ΔCt trends over time among specimen types at each temperature (Fig. 2D through F; Table S2). Across storage conditions, ΔCt values increased over time for all specimen types, with the magnitude of change varying by temperature. At 4°C, unpreserved stool demonstrated a modest but significant increase in ΔCt (≈0.15 Ct/day, *P* = 0.013), eSwab showed a similar rate of change (*P* = 0.82 vs Neat), and FecalSwab exhibited a significantly slower increase (*P* = 0.046), indicating improved stability. At room temperature, unpreserved samples again showed a significant increase (≈0.22 Ct/day, *P* = 0.005), while eSwab and FecalSwab did not differ significantly from unpreserved stool in baseline or slope, although FecalSwab showed a non-significant trend toward slower degradation. At 37°C, unpreserved samples began at a higher baseline ΔCt (~2.2 Ct) and increased more rapidly over time (≈0.48 Ct/day, *P* < 0.001), with no significant differences observed between swab types and unpreserved stool. Overall, FecalSwab and eSwab specimens demonstrated stability comparable to unpreserved stool across temperatures, with FecalSwab showing a tendency toward enhanced stability at lower and room temperatures.

### Performance characteristics of BD MAX Cdiff assay on FecalSwab and eSwab specimens compared with neat stool

The sensitivity of the BD MAX Cdiff assay using 100 µL of FecalSwab specimen compared with 10 µL of unpreserved stool was 98.5% (95% CI: [91.7–100.0]) with a specificity of 98.4% (95% CI: [91.3–100.0]). The overall accuracy was 98.4% (95% CI: [94.4–99.8]) with a kappa coefficient (agreement beyond chance) of 0.968 (*P* < 0.001). The sensitivity of BD MAX Cdiff assay using 100 µL of eSwab specimen compared to 10 µL of unpreserved stool was 97.6% (95% CI: [87.4–99.9]) with a specificity of 100.0% (95% CI: [93.0–100.0]). The accuracy was 98.9% (95% CI: [94.2–100.0]) with a kappa coefficient of 0.978 (*P* < 0.001) (Table 2). When comparing 100 µL of eSwab specimen with FecalSwab specimen, the percent agreement was 98.4% (95% CI: [95.2–100.0]) (Table 3). A total of two samples yielded discordant results among specimen types. One sample was positive in unpreserved stool (Ct = 31.9) but negative by both swab tests, whereas the other sample was positive only in the FecalSwab specimen (Ct = 33.3). No repeat testing was performed.

**TABLE 2:** Performance characteristics of BD MAX Cdiff assay on FecalSwab and eSwab specimens compared with neat stool

Statistics	FecalSwab vs neat stool (*N* = 127)		eSwab vs neat stool (*N* = 93)	
	Value	95% CI	Value	95% CI
True positive	64	N/A^*a*^	41	N/A
False positive	1	N/A	0	N/A
True negative	61	N/A	51	N/A
False negative	1	N/A	1	N/A
Sensitivity	98.5%	91.7–100.0%	97.6%	87.4–99.9%
Specificity	98.4%	91.3–100.0%	100.0%	93.0–100.0%
Accuracy	98.4%	94.4–99.8%	98.9%	94.2–100.0%
Kappa	0.968	0.946–0.990, *P* < 0.001	0.978	0.956–1.000, *P* < 0.001

^*a*^ N/A, not applicable.

**TABLE 3:** Agreement between BD MAX Cdiff test results obtained from FecalSwab and eSwab specimens

Fecal Swab vs eSwab		eSwab		Total
		POS	NEG	
FecalSwab	POS	28	1	29
	NEG	0	32	32
Total		28	33	61
Percent agreement		98.4% (95% CI [95.2–100])		

## DISCUSSION

There is currently a wide range of molecular testing options available for the detection of *C. difficile,* including commercially available and lab-developed tests. As patients with suspected CDI are placed on pre-emptive contact precautions pending test results (2), timely diagnosis of CDI is of importance for infection control and patient management. The BD MAX Cdiff assay provides rapid detection of the *C. difficile* toxin B gene with high sensitivity (12), decreasing the time to diagnosis compared to traditional methods such as toxigenic culture. The BD MAX Cdiff PCR assay is a fully automated solution with limited hands-on time, and results are typically available in as little as 2 h. Unpreserved stool remains the only Health Canada- and FDA-approved specimen type for this assay, but swabs collected from stool and contained in an appropriate medium could allow for easier transport and improved specimen preservation for *C. difficile* testing. Comparison of unpreserved stool and swabs collected from stool in this study shows that FecalSwab and eSwab media could be an acceptable alternative to unpreserved stool for use in the BD MAX Cdiff assay. As equivalent performance has already been demonstrated using FecalSwab specimens on the BD MAX Enteric Bacterial and Enteric Viral panels (13, 14), this approach could be easily adapted into existing testing algorithms using the BD MAX platform.

Verification using varying volumes of FecalSwab and eSwab media was performed initially, and while all volumes tested showed good concordance with the manufacturer's recommended testing of unpreserved stool, 100 uL was selected as the optimal volume of both media based on replicability, negligible change in Ct values compared to larger specimen volumes, and lab workflow considerations. Previously, 50 and 70 µL of FecalSwab medium were used in the BD MAX assay (9, 10), and 200 µL of FecalSwab medium was used in the Xpert *C. difficile* assay (8). Thus, our optimal volume of FecalSwab medium falls within the estimated range of specimen volumes reported in the literature. To our knowledge, eSwabs collected from stool specimens have not yet been evaluated for use on the BD MAX Cdiff assay nor have they been directly compared to FecalSwab medium on the BD MAX Cdiff assay.

In this study, we demonstrate that swabs of stool collected into FecalSwab and eSwab media have good agreement with unpreserved stool for the detection of *C. difficile* using the BD MAX Cdiff PCR assay. The sensitivity of eSwab medium was slightly lower when compared with FecalSwab (97.6% vs 98.5%) but overall, both FecalSwab and eSwab demonstrated high sensitivity. Furthermore, swabs of stool collected into FecalSwab and eSwab media showed strong agreement with each other (98.4%). In all discordant results, the Ct values were over 30 and likely nearing the assay cutoff. One limitation of these experiments is the differing sample sizes between the three sets of comparisons. Initial experiments were conducted using only FecalSwab specimens, but eSwab was added to the study due to its frequent use in hospital settings, resulting in different sample sizes.

When comparing the stability of FecalSwab and eSwab specimens to unpreserved stool over 14 days under varying storage conditions, both swab types maintained *C. difficile* DNA stability comparable to unpreserved stool across all temperatures tested. Although ΔCt values increased over time, most notably at 37°C, the absence of significant differences among specimen types at individual time points supports the analytical equivalence of swab-based collection systems for testing on the BD MAX Cdiff assay. While clinical samples are rarely transported or stored at 37°C, this temperature may be relevant in resource-limited settings with warmer climates. Although all samples remained positive at 37°C for 14 days in our study, weakly positive specimens with low bacterial loads could be more affected under these conditions. FecalSwab specimens consistently demonstrated the smallest changes in Ct values over time, with a significantly slower rate of increase at 4°C and a similar trend at room temperature, suggesting a modest stabilizing effect of the transport medium under conditions typical of clinical transport and short-term storage.

Overall, swabs of stool collected into FecalSwab and eSwab media could be acceptable alternative specimen types for use with the BD MAX Cdiff PCR assay. FecalSwab and eSwab collection kits contain flocked swabs and pre-filled tubes, allowing healthcare providers or patients to collect a specimen directly from a brief or stool collection hat, eliminating the need for transfer spatulas used for neat stool collection. This approach simplifies specimen acquisition and may improve consistency. Within the laboratory, all materials required for the BD MAX Cdiff PCR assay are supplied in the testing kits, and staff are required to perform only a single specimen transfer using a standard laboratory pipette, thereby reducing technical workload. In rural and remote settings where specimen transport and stability may pose challenges, stool swabs collected in FecalSwab or eSwab media could serve as valuable alternatives, facilitating easier collection and accommodating longer transport intervals.

## SUPPLEMENTAL MATERIAL

Supplemental material spectrum.00767-26-s0001.docx Tables S1 and S2.

ASM does not own the copyrights to Supplemental Material that may be linked to, or accessed through, an article. The authors have granted ASM a non-exclusive, world-wide license to publish the Supplemental Material files. Please contact the corresponding author directly for reuse.

## References

[1] Czepiel J, Dróżdż M, Pituch H, Kuijper EJ, Perucki W, Mielimonka A, Goldman S, Wultańska D, Garlicki A, Biesiada G. 2019. Clostridium difficile infection: review. Eur J Clin Microbiol Infect Dis 38:1211–1221. doi:10.1007/s10096-019-03539-6 30945014 PMC6570665

[2] McDonald LC, Gerding DN, Johnson S, Bakken JS, Carroll KC, Coffin SE, Dubberke ER, Garey KW, Gould CV, Kelly C, Loo V, Shaklee Sammons J, Sandora TJ, Wilcox MH. 2018. Clinical practice guidelines for Clostridium difficile infection in adults and children: 2017 update by the Infectious Diseases Society of America (Idsa) and Society for Healthcare Epidemiology of America (Shea). Clin Infect Dis 66:e1–e48. doi:10.1093/cid/cix1085 29462280 PMC6018983

[3] CDC. 2024. Clinical testing and diagnosis for C. diff Infection. https://www.cdc.gov/c-diff/hcp/diagnosis-testing/index.html.

[4] Gateau C, Couturier J, Coia J, Barbut F. 2018. How to: diagnose infection caused by Clostridium difficile. Clin Microbiol Infect 24:463–468. doi:10.1016/j.cmi.2017.12.005 29269092

[5] Tenover FC, Baron EJ, Peterson LR, Persing DH. 2011. Laboratory diagnosis of Clostridium difficile infection can molecular amplification methods move us out of uncertainty? J Mol Diagn 13:573–582. doi:10.1016/j.jmoldx.2011.06.001 21854871 PMC3194048

[6] Azrad M, Tkhawkho L, Hamo Z, Peretz A. 2020. The diagnostic performance and accuracy of 3 molecular assays for the detection of Clostridium difficile in stool samples, compared with the Xpert C. difficile assay. J Microbiol Methods 168:105784. doi:10.1016/j.mimet.2019.105784 31758952

[7] Shin BM, Yoo SM, Shin WC. 2016. Evaluation of Xpert C. difficile, BD MAX Cdiff, IMDx C. difficile for Abbott m2000, and Illumigene C. difficile assays for direct detection of toxigenic Clostridium difficile in stool specimens. Ann Lab Med 2:131–137. doi:10.3343/alm.2016.36.2.131 PMC4713846 26709260

[8] Mashock MJ, Faron ML, Buchan BW, Ledeboer NA. 2017. Evaluation of Copan fecalSwab as specimen type for use in Xpert C. difficile assay. J Clin Microbiol 55:3123–3129. doi:10.1128/JCM.00369-17 28794179 PMC5625397

[9] Hirvonen JJ, Kaukoranta SS. 2015. Comparison of BD Max Cdiff and GenomEra C. difficile molecular assays for detection of toxigenic Clostridium difficile from stools in conventional sample containers and in FecalSwabs. Eur J Clin Microbiol Infect Dis 34:1005–1009. doi:10.1007/s10096-015-2320-2 25616552

[10] Anne-Gaëlle R, Célia S, Jeanne C, Frédéric B, Anne T, Coralie B, Kevin S, Sonia F, Pascaline D, Francois V, Olivier D, Fréderic L. 2022. Performances of the BD MAX CDIFF assay for the detection of toxigenic Clostridioides difficile using cary-blair preserved samples. Diagn Microbiol Infect Dis 103:115701. doi:10.1016/j.diagmicrobio.2022.115701 35596982

[11] Hirvonen JJ, Kaukoranta SS. 2014. Comparison of FecalSwab and ESwab devices for storage and transportation of diarrheagenic bacteria. J Clin Microbiol 52:2334–2339. doi:10.1128/JCM.00539-14 24740083 PMC4097757

[12] Putsathit P, Morgan J, Bradford D, Engelhardt N, Riley TV. 2015. Evaluation of the BD Max Cdiff assay for the detection of toxigenic Clostridium difficile in human stool specimens. Pathology (Phila) 47:165–168. doi:10.1097/PAT.0000000000000214 25551308

[13] Richard-Greenblatt M, Rutherford C, Luinstra K, Cárdenas AM, Pang XL, Jayaratne P, Smieja M. 2020. Evaluation of the fecalswab for stool specimen storage and molecular detection of enteropathogens on the BD max system. J Clin Microbiol 58:10. doi:10.1128/JCM.00178-20 PMC7448620 32461284

[14] Rojas HF, Lima A, Kubasek C, Gostnell A, Silbert S. 2020. Evaluation of Copan FecalSwab preserved stool specimens with the BD MAX enteric bacterial panel and the BD MAX extended enteric bacterial panel. Diagn Microbiol Infect Dis 97:115055. doi:10.1016/j.diagmicrobio.2020.115055 32470844

