# Supplementary material for: Evaluation of COPAN FecalSwab and eSwab collection systems for the detection of Clostridioides difficile using the BD MAX Cdiff assay
Source: Microbiol Spectr. 2026 May 1;14(6):e00767-26. doi: 10.1128/spectrum.00767-26 (PMC13228037; doi:10.1128/spectrum.00767-26)
Supplement: Supplemental material — Tables S1 and S2. [file spectrum.00767-26-s0001.docx]

**Supplemental Material**

Supplemental Table 1 (S1): One-Way ANOVA results comparing delta Cts between specimen type at each timepoint and temperature condition.

| Condition | F (2,6) | P value | Levene p value |
| --- | --- | --- | --- |
| 4C, Day 2 | 2.814 | 0.137 | 0.191 |
| 4C, Day 7 | 2.063 | 0.208 | 0.912 |
| 4C, Day 14 | 2.870 | 0.133 | 0.549 |
| RT, Day 2 | 0.460 | 0.652 | 0.069 |
| RT, Day 7 | 0.491 | 0.635 | 0.771 |
| RT, Day 14 | 0.912 | 0.451 | 0.160 |
| 37C, Day 2 | 2.009 | 0.215 | 0.417 |
| 37C, Day 7 | 1.905 | 0.229 | 0.601 |
| 37C, Day 14 | 1.471 | 0.302 | 0.700 |

**Supplemental Table 2 (S2): Linear Regression Analysis of deltaCT by Day and Swab Type Across Temperatures (4°C, Room Temperature, and 37°C)**

| **Temperature** | **Term** | **Estimate** | **Std. Error** | **t value** | **p-value** | **Significance** |
| --- | --- | --- | --- | --- | --- | --- |
| 4°C | (Intercept) | 0.0597 | 0.4434 | 0.135 | 0.8938 |  |
|  | day | 0.1534 | 0.0562 | 2.73 | 0.0105 | * |
|  | swab_typeeSwab | -0.24 | 0.627 | -0.383 | 0.7045 |  |
|  | swab_typeFecalSwab | -0.5683 | 0.627 | -0.906 | 0.372 |  |
|  | day:swab_typeeSwab | 0.0186 | 0.0795 | 0.234 | 0.8169 |  |
|  | day:swab_typeFecalSwab | -0.1664 | 0.0795 | -2.094 | 0.0448 | * |
|  | Residual SE | 1.052 |  |  |  |  |
|  | Adjusted R² | 0.4386 |  |  |  |  |
|  | F-statistic (df=5,30) | 6.47 |  |  | 0.0003 | *** |
| RT | (Intercept) | 0.1701 | 0.5592 | 0.304 | 0.7631 |  |
|  | day | 0.2154 | 0.0709 | 3.039 | 0.0049 | ** |
|  | swab_typeeSwab | 0.0997 | 0.7908 | 0.126 | 0.9005 |  |
|  | swab_typeFecalSwab | 0.1337 | 0.7908 | 0.169 | 0.8669 |  |
|  | day:swab_typeeSwab | -0.0985 | 0.1002 | -0.983 | 0.3336 |  |
|  | day:swab_typeFecalSwab | -0.1769 | 0.1002 | -1.765 | 0.0878 | . |
|  | Residual SE | 1.326 |  |  |  |  |
|  | Adjusted R² | 0.2207 |  |  |  |  |
|  | F-statistic (df=5,30) | 2.982 |  |  | 0.0266 | * |
| 37°C | (Intercept) | 2.2141 | 0.9808 | 2.258 | 0.0314 | * |
|  | day | 0.4758 | 0.1243 | 3.828 | 0.0006 | *** |
|  | swab_typeeSwab | -1.8227 | 1.387 | -1.314 | 0.1988 |  |
|  | swab_typeFecalSwab | -1.445 | 1.387 | -1.042 | 0.3058 |  |
|  | day:swab_typeeSwab | -0.0975 | 0.1758 | -0.555 | 0.5833 |  |
|  | day:swab_typeFecalSwab | -0.2139 | 0.1758 | -1.217 | 0.2331 |  |
|  | Residual SE | 2.326 |  |  |  |  |
|  | Adjusted R² | 0.4845 |  |  |  |  |
|  | F-statistic (df=5,30) | 7.58 |  |  | 0.0001 | *** |
